# Improved positive predictive value of non-invasive prenatal testing through integration with second-trimester ultrasound soft markers for fetal chromosomal abnormalities: a retrospective cohort study

**DOI:** 10.3389/fendo.2026.1831332

**Published:** 2026-05-29

**Authors:** Dan Fu, Qian Wu, Yun Ju, Min Jiang, Suhua Zhang

**Affiliations:** 1 Department of Prenatal Diagnosis, Northern Jiangsu People’s Hospital Affiliated to Yangzhou University, Yangzhou, Jiangsu, China; 2 Molecular Diagnostics Laboratory, Northern Jiangsu People’s Hospital Affiliated to Yangzhou University, Yangzhou, Jiangsu, China

**Keywords:** cell-free fetal DNA, confined placental mosaicism, fetal aneuploidy, non-invasive prenatal testing, positive predictive value, prenatal diagnosis, risk stratification, trisomy 21

## Abstract

**Background:**

Non-invasive prenatal testing (NIPT) achieves high sensitivity for common fetal aneuploidies; however, its positive predictive value (PPV) varies substantially with population prevalence, generating a considerable false-positive burden. Current guidelines offer limited guidance on ultrasound-based risk refinement after a positive NIPT result.

**Objective:**

To determine whether integrating second-trimester ultrasound soft markers with NIPT improves PPV for fetal chromosomal abnormalities and to characterize aneuploidy-type-specific phenotypic profiles to inform risk stratification.

**Methods:**

This retrospective cohort study included 303 singleton pregnancies at a Chinese tertiary center (September 2022–September 2025). All patients underwent NIPT (12+0-14+6 weeks) and detailed second-trimester ultrasound (19–23 weeks). Eight soft marker categories were assessed. Chromosomal abnormalities were confirmed by karyotyping, and neonatal phenotypic assessment served as the reference for NIPT-negative pregnancies. Predefined combined strategies were compared using diagnostic accuracy metrics and receiver operating characteristic analysis.

**Results:**

Twenty-four pregnancies (7.9%) harbored confirmed chromosomal abnormalities. NIPT achieved 100% sensitivity and 100% negative predictive value (upper-bound estimates given differential verification bias; see Methods), but only 55.8% PPV (24/43), with 19 false positives. Among the false-positive cases, 63.2% exhibited no soft markers, and none had ≥2 markers. The high-risk combined strategy (NIPT-positive with ≥1 high-risk marker or ≥2 markers) improved PPV to 83.3% with 98.9% specificity, potentially reducing invasive procedures by 58% through individualized counseling. Strategy (a) (NIPT-positive with ≥ 1 marker) achieved 100% sensitivity for trisomies 18 and 13 (uniformly severe marker burdens) but 62.5% for trisomy 21 (37.5% marker-negative). The combined model achieved an AUC of 0.987, significantly exceeding that of NIPT alone (0.966; p = 0.012), although these estimates warrant external validation given the enriched cohort. Women aged <35 years derived the greatest incremental benefit (+28.2 percentage points).

**Conclusions:**

In this enriched tertiary referral cohort, integrating second-trimester ultrasound soft markers with NIPT was associated with improved PPV, with particular efficacy for trisomies 18 and 13; however, the observed PPV improvement was predominantly driven by trisomies 18 and 13, which uniformly present with multiple markers, whereas the clinical utility for trisomy 21 was more limited owing to phenotypic heterogeneity (37.5% marker-negative). If confirmed by prospective multicenter validation, this approach may enable individualized post-positive NIPT counseling and complement existing guideline frameworks.

## 1 Introduction

Chromosomal aneuploidies, principally trisomies 21, 18, and 13, remain among the most prevalent causes of perinatal morbidity, neurodevelopmental disability, and pregnancy loss, collectively affecting approximately 1 in 150 live births (1–3). The imperative for reliable prenatal detection extends beyond the diagnosis itself; it underpins informed reproductive decision-making, anticipatory obstetric management, and timely genetic counseling for affected families.

Non-invasive prenatal testing (NIPT) based on cell-free fetal DNA (cffDNA) analysis has fundamentally transformed prenatal screening since its clinical introduction in 2011 (4, 5), offering substantial improvements over prior sequential and combined first-trimester screening approaches (6, 7) and contributing to the rapid global expansion of cell-free DNA-based screening (8). Meta-analytic evidence consistently demonstrates sensitivities exceeding 99% and specificities exceeding 99.5% for trisomy 21, with a comparably robust performance for trisomies 18 and (9–11), findings further corroborated by large prospective trials comparing cfDNA with conventional screening (12). Professional societies, including the American College of Obstetricians and Gynecologists (ACOG), the Society for Maternal-Fetal Medicine (SMFM), and the International Society for Prenatal Diagnosis (ISPD), uniformly recommend offering cfDNA screening for common aneuploidies to all pregnant individuals (13–15). Despite these credentials, the clinical utility of any screening test ultimately hinges on its positive predictive value (PPV), a parameter governed not only by intrinsic test accuracy but also by disease prevalence in the population being screened (16, 17). In practice, NIPT PPV fluctuates considerably from approximately 45% to 95% according to maternal age, fetal fraction, and cohort composition (18, 19). This variability translates into a non-negligible false-positive burden, each positive result potentially precipitating invasive diagnostic procedures that carry a procedure-related pregnancy loss risk of 0.1–0.3% (20, 21).

The second-trimester detailed ultrasound (18–24 weeks) offers an independent phenotypic window into fetal chromosomal status. Non-structural sonographic findings designated “soft markers” echogenic intracardiac focus (EIF), choroid plexus cysts (CPC), pyelectasis, increased nuchal fold thickness, shortened long bones, echogenic bowel, and hypoplastic nasal bone demonstrate well-established associations with aneuploidy (22–25). The SMFM Consult Series #57 provides contemporary guidance on isolated soft marker management, concluding that diagnostic testing is not warranted solely for an isolated marker following reassuring cfDNA results (15). However, the converse clinical scenario whether ultrasound findings can meaningfully refine risk after a positive NIPT result remains systematically underexplored. Spingler et al. (26) demonstrated that the PPV for trisomy 18 or 13 following a high-risk cffDNA result declined to 9.5% when ultrasound was normal yet reached 100% when abnormalities were identified. Wan et al. (27) reported that cffDNA screening combined with second-trimester soft markers yielded a PPV of 71.4% for common trisomies in a Chinese cohort. Chen et al. (28), in a comparable retrospective study of 306 pregnancies, found that standard NIPT combined with ultrasound achieved 88.89% PPV for common trisomies. These observations collectively suggest that ultrasound serves as an effective adjunct for discriminating true fetal aneuploidy from confined placental mosaicism (CPM) (29, 30), yet no study has systematically compared predefined combined strategies or characterized aneuploidy-type-specific ultrasound profiles within a unified analytical framework.

In China, the rapid adoption of NIPT across multiple domestic platforms (31, 32) and the increasing proportion of pregnancies among women of advanced maternal age following family planning policy reforms (33, 34) have intensified the need for screening strategies that preserve detection rates while reducing false-positive burdens. Detailed second-trimester ultrasonography is already universally performed as routine prenatal care in China, providing an existing infrastructure for combined interpretation without additional cost. Accordingly, the present study was designed to evaluate the diagnostic performance of NIPT and second-trimester ultrasound soft markers, individually and in combination, for fetal chromosomal abnormality detection; to characterize aneuploidy-type-specific phenotypic marker profiles; and to define risk-stratified integration strategies for the post-positive NIPT clinical scenario.

## 2 Materials and methods

### 2.1 Study design and setting

This retrospective observational cohort study was conducted at the Department of Prenatal Diagnosis, Northern Jiangsu People’s Hospital Affiliated to Yangzhou University, a tertiary referral center serving approximately 5 million residents in northern Jiangsu, China. The department functions as the primary regional referral center for NIPT-positive cases, abnormal first-trimester screening, advanced maternal age pregnancies, and self-referred patients requesting enhanced screening. Detailed second-trimester ultrasound is provided to all pregnancies managed at this center as part of routine care, consistent with Chinese national prenatal care guidelines. Medical records were reviewed for all consecutive eligible pregnancies during the 36-month period from September 1, 2022, to September 30, 2025. The resulting cohort represents all pregnancies that completed both NIPT and second-trimester ultrasound during this period rather than a selected subset; the elevated aneuploidy prevalence relative to unselected populations reflects the tertiary referral composition of the center. The study was approved by the Institutional Ethics Committee. The requirement for informed consent was waived owing to the retrospective design and use of de-identified data. All procedures were conducted in accordance with the principles of the Declaration of Helsinki. Reporting followed the Strengthening the Reporting of Observational Studies in Epidemiology (STROBE) guidelines (35).

### 2.2 Study population

Eligible pregnancies were identified through cross-referencing of prenatal diagnosis department databases, NIPT laboratory records, and ultrasound department logs. The inclusion criteria were as follows: singleton pregnancy, maternal age ≥ 18 years, completion of NIPT between 12+0 and 14+6 weeks gestation, completion of a detailed second-trimester ultrasound between 19^+0^ and 23^+6^ weeks’ gestation, and availability of complete pregnancy outcome data. The exclusion criteria were as follows: multiple gestation, major fetal structural anomalies identified on ultrasound necessitating separate genetic evaluation (e.g., congenital heart defects, neural tube defects), NIPT test failure or fetal fraction < 4%, incomplete ultrasound examination or absent soft marker documentation, loss to follow-up prior to outcome determination, maternal malignancy or organ transplantation (potential sources of non-fetal cffDNA), and incomplete medical records.

### 2.3 NIPT procedures

Blood samples (10 mL) were collected in EDTA tubes at 12+0–14+6 weeks’ gestation. Cell-free DNA was extracted from maternal plasma using commercially available kits. NIPT was performed using one of three validated platforms based on patient preference and insurance coverage: BGI-NIFTY (BGI Genomics, Shenzhen, China), Berry NIPT-seq (Berry Genomics, Beijing, China), or Harmony (Roche Diagnostics, Basel, Switzerland). All platforms employed massively parallel sequencing with proprietary bioinformatics algorithms. Z-scores were calculated for chromosomes 21, 18, 13, and the sex chromosomes, with values ≥ 3.0 designated as positive for autosomal chromosomes; platform-specific thresholds were applied for sex chromosome analysis in accordance with each manufacturer’s validated algorithm. Results were classified as positive, negative, or not applicable (test failure). A 4% fetal fraction threshold was selected in accordance with the manufacturer-validated analytical specifications for all three platforms, below which the signal-to-noise ratio is insufficient for reliable aneuploidy detection; this cutoff is consistent with the threshold recommended by the American College of Medical Genetics and Genomics (ACMG) evidence-based guidelines for NIPT (14) and with the commonly accepted quality threshold described in the international literature (29, 30). Fetal fraction and test quality metrics were routinely recorded; however, individual-level fetal fraction data were not uniformly available across all two platforms in our archived records, precluding a formal comparative analysis of fetal fraction between true-positive and false-positive cases.

### 2.4 Second-trimester ultrasound examination

Detailed ultrasound examinations were performed by maternal-fetal medicine specialists (> 5 years’ experience, > 2,000 examinations) using high-resolution systems (GE Voluson E10; Samsung WS80A). Each examination comprised a comprehensive anatomical survey and systematic soft marker assessment in accordance with the updated International Society of Ultrasound in Obstetrics and Gynecology (ISUOG) Practice Guidelines (36). The following eight soft markers were evaluated using standardized criteria: EIF (bright echogenic focus in the ventricle with echogenicity equal to bone), CPC (anechoic cysts > 3 mm in the choroid plexus), pyelectasis (anteroposterior renal pelvis diameter ≥ 4 mm before 24 weeks), increased nuchal fold (≥ 6 mm at 18–24 weeks), short femur (< 5th percentile for gestational age), short humerus (< 5th percentile for gestational age), echogenic bowel (echogenicity equal to or exceeding adjacent bone), and hypoplastic nasal bone (absent on mid-sagittal profile view or nasal bone length < 2.5 mm). Each fetus was classified according to the total marker count (0, 1, 2, or ≥ 3). Single umbilical artery and mild ventriculomegaly, recognized as soft markers in certain classification systems, including SMFM Consult #57 (15), were not included: single umbilical artery was not systematically recorded in the study database during the early portion of the study period, and mild ventriculomegaly was classified under structural anomalies at our institution. These omissions represent a limitation of the marker assessment.

### 2.5 Definition of combined screening strategies and marker risk classification

To evaluate the incremental value of ultrasound findings in the context of positive NIPT results, four predefined combined screening strategies were assessed. These strategies were designed to span a spectrum of clinical stringency from least restrictive (any marker) to most restrictive (≥ 2 markers), with two intermediate strategies incorporating marker risk classification, thereby enabling a systematic evaluation of the sensitivity–PPV trade-off. Soft markers were classified *a priori* into two categories based on established literature regarding their association strength with aneuploidy (23, 37, 38) and consistent with the likelihood ratio–based hierarchy described in SMFM Consult #57 (15): high-risk markers, comprising increased nuchal fold (≥ 6 mm), short femur, short humerus, echogenic bowel, and hypoplastic nasal bone; and low-risk markers, comprising isolated EIF, isolated CPC, and isolated pyelectasis. The four combined strategies were as follows: (a) NIPT positive AND ≥ 1 soft marker of any type; (b) NIPT positive AND ≥ 2 soft markers of any type; (c) NIPT positive AND presence of ≥ 1 high-risk marker or ≥ 2 markers of any type (hereafter termed the “high-risk combined strategy”); and (d) NIPT positive OR ≥ 2 markers (a parallel screening approach designed as a theoretical sensitivity benchmark by designating a pregnancy as screen-positive if either criterion is met independently; this strategy was included to define the maximum achievable detection rate and to illustrate the sensitivity–PPV trade-off, rather than as a practical clinical recommendation given its substantially reduced PPV).

### 2.6 Genetic counseling and invasive diagnostic testing

Genetic counseling was provided for pregnancies with positive NIPT results, ≥ 2 soft markers, advanced maternal age (≥ 35 years) with any screening abnormality, or at the patient ‘s request. Amniocentesis was performed at ≥ 16 weeks’ gestation using an ultrasound-guided transabdominal technique. No procedure-related pregnancy losses were observed; however, the study was not powered to evaluate procedural complication rates. Fetal karyotyping was conducted using G-banding at 400–550 band resolution. Chromosomal microarray analysis (CMA) was performed on selected cases using single-nucleotide polymorphism (SNP) arrays.

### 2.7 Reference standard and case classification

The reference standard for chromosomal status was fetal karyotyping for all pregnancies that underwent invasive testing and neonatal phenotypic assessment for pregnancies classified as low-risk that did not undergo invasive procedures. All 43 NIPT-positive pregnancies underwent amniocentesis and karyotyping. Among the 260 NIPT-negative pregnancies, a subset underwent amniocentesis for indications other than NIPT positivity (e.g., ≥ 2 soft markers, advanced maternal age with additional risk factors, or patient request), all yielding normal karyotypes; the remainder were classified based on neonatal phenotypic assessment at birth and pediatric follow-up. Pregnancies were designated as true positives (TP), positive NIPT confirmed by an abnormal karyotype; false positives (FP), positive NIPT with a normal karyotype; true negatives (TN), negative NIPT with a normal karyotype or normal neonatal phenotype; and false negatives (FN), negative NIPT with a subsequently confirmed chromosomal abnormality.

This differential verification design, in which the reference standard differs between screen-positive and screen-negative groups, introduces verification bias that may inflate sensitivity and negative predictive value (NPV) estimates. Neonatal phenotypic assessment has limited capacity to detect mosaic trisomies, low-level mosaicism, and certain sex chromosome aneuploidies (39). Therefore, the reported 100% sensitivity and 100% NPV should be interpreted as upper-bound estimates. This limitation is inherent to virtually all NIPT screening studies that do not employ universal karyotyping and is acknowledged as a potential source of bias throughout the interpretation of results.

### 2.8 Data collection and quality control

Data were extracted from electronic medical records by trained research personnel using a standardized collection form. The information collected included maternal demographics (age, BMI, ethnicity, gravidity, and parity), NIPT parameters (platform, gestational age, Z-scores, fetal fraction, and result), ultrasound findings (gestational age, individual marker presence/absence, and total marker count), genetic testing results, and pregnancy outcomes. Two independent reviewers extracted data from a randomly selected 10% subset; discrepancies were resolved by consensus. Inter-rater reliability was quantified using Cohen’s kappa coefficient. The principal investigator independently reviewed all confirmed chromosomal abnormality cases.

### 2.9 Statistical analysis

Analyses were performed using SPSS version 26.0 (IBM Corporation) and R version 4.2.0 (packages: logistf (40) for Firth’s penalized logistic regression; pROC (41) for ROC analysis and DeLong test). Continuous variables are presented as mean ± standard deviation (SD) or median (interquartile range), depending on the normality of the distribution, as assessed by the Shapiro–Wilk test. Categorical variables are expressed as frequencies and percentages. Group comparisons used the chi-square test or Fisher’s exact test for categorical data and the independent-samples t-test or Mann–Whitney U test for continuous data.

Diagnostic performance metrics (sensitivity, specificity, PPV, NPV, and likelihood ratios) were calculated for NIPT alone, ultrasound markers alone, and each combined strategy. Exact binomial 95% confidence intervals (CI) were computed for all proportions. Multivariable logistic regression was performed to identify independent predictors of chromosomal abnormalities. Given the relatively small number of outcome events (n = 24), the model was restricted to three candidate variables (NIPT result, number of soft markers, and maternal age) to maintain an adequate events-per-variable ratio. Because NIPT yielded no false-negative results (producing quasi-complete separation), Firth’s penalized maximum likelihood estimation was employed to obtain finite, bias-corrected odds ratio estimates (42). The resulting odds ratio for NIPT positivity represents a boundary estimate influenced by quasi-complete separation and should be interpreted as indicating the direction and strength of association rather than a precise quantitative effect. Receiver operating characteristic (ROC) curves were constructed for each strategy, and area under the curve (AUC) values were compared using the DeLong test, which accounts for the correlation between paired ROC curves derived from the same sample (43). A two-sided p < 0.05 was considered statistically significant. Bonferroni correction was applied for the comparison of eight individual soft markers with chromosomal status (adjusted significance threshold: p < 0.00625) and for pairwise comparisons among the four combined strategies. Subgroup analyses by maternal age and aneuploidy type were considered exploratory and were not adjusted for multiplicity.

### 2.10 Sample size considerations

Given the retrospective design of the study, all eligible cases were included during the study period. No *a priori* sample-size calculations were performed.

## 3 Results

### 3.1 Study population

**Figure 1:**
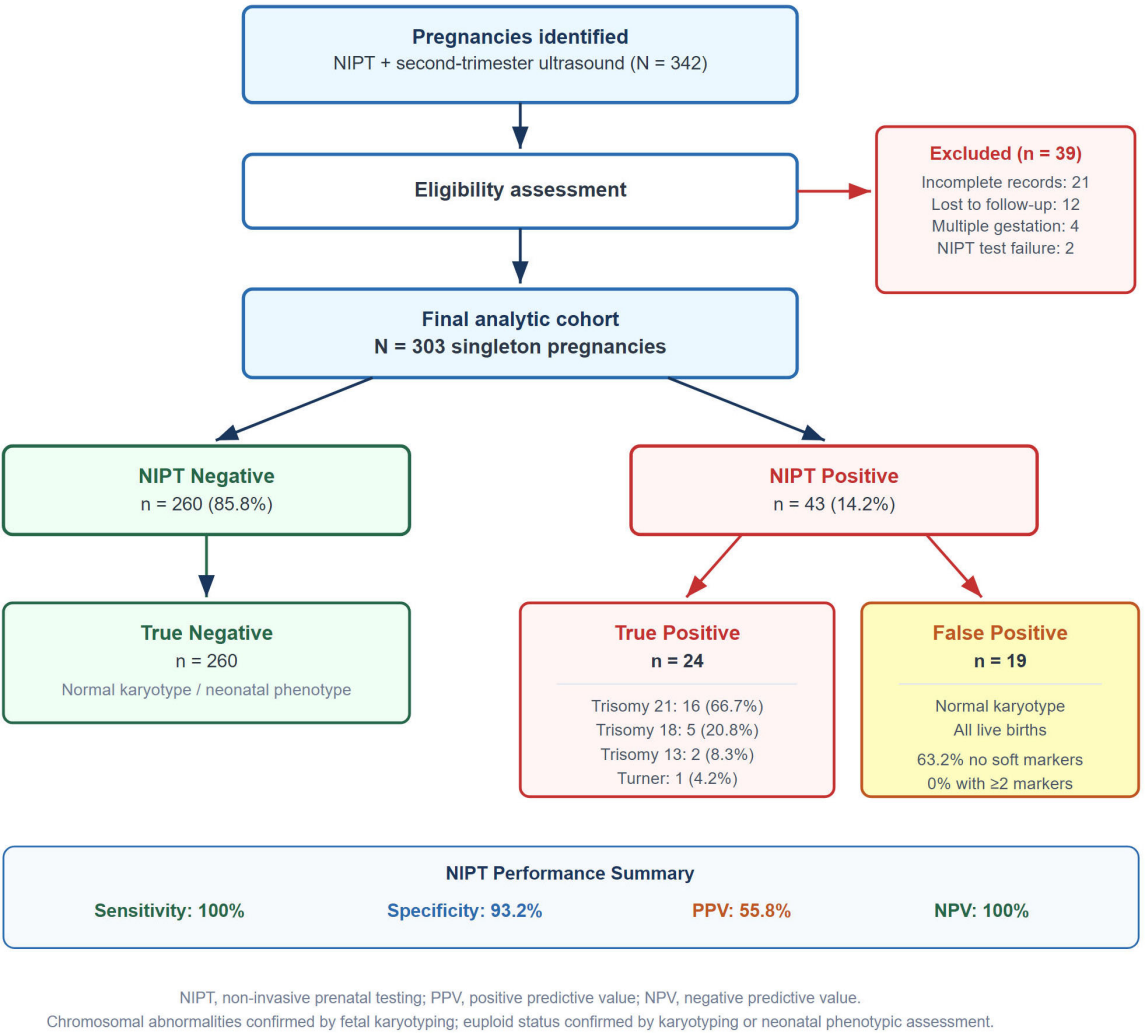
Study flow diagram. Of 342 initially identified pregnancies, 303 met inclusion criteria. NIPT detected 43 positive results: 24 true positives confirmed by karyotyping and 19 false positives with normal karyotypes. All 260 NIPT-negative cases were confirmed as true negatives by karyotyping or neonatal phenotypic assessment.

Medical records of 342 pregnancies that underwent both NIPT and second-trimester ultrasound examinations were identified during the study period. After applying the exclusion criteria, 303 pregnancies constituted the final cohort (**Figure 1**). The reasons for exclusion were as follows: incomplete medical records (n = 21), loss to follow-up (n = 12), multiple gestation (n = 4), and NIPT test failure (n = 2). No missing data were present for the primary variables among the included pregnancies. The inter-rater reliability for data extraction yielded a Cohen’s kappa of 0.94 (95% CI: 0.88–1.00), indicating excellent agreement.

Demographic and clinical characteristics, stratified by chromosomal outcome, are summarized in **Table 1**. The mean maternal age was 31.2 ± 4.2 years; 64 women (21.1%) were of advanced maternal age (≥ 35 years). The population was predominantly Han Chinese (91.1%). The most common indication for NIPT was patient request or routine screening (57.4%), followed by advanced maternal age (19.1%). NIPT was performed using either the CapitalBio/Boao Proton platform or the BGI MGISEQ-200 sequencing platform. A second-trimester ultrasound was performed at a mean of 20.8 ± 1.2 weeks.

**Table 1:** Demographic and clinical characteristics (N = 303).

Characteristic	Overall (N = 303)	Aneuploid (n = 24)	Euploid (n = 279)	p-value
Maternal age, years	31.2 ± 4.2	37.4 ± 3.8	30.4 ± 3.8	< 0.001
Age ≥ 35 years, n (%)	64 (21.1)	18 (75.0)	46 (16.5)	< 0.001
BMI, kg/m²	23.8 ± 3.2	25.2 ± 3.8	23.7 ± 3.1	0.057
Han Chinese ethnicity, n (%)	276 (91.1)	22 (91.7)	254 (91.0)	1.000
Nulliparous, n (%)	151 (49.8)	11 (45.8)	140 (50.2)	0.688
GA at NIPT, weeks	12.6 ± 0.8	12.4 ± 0.7	12.6 ± 0.8	0.258
GA at ultrasound, weeks	20.8 ± 1.2	20.6 ± 1.3	20.8 ± 1.2	0.463
Primary NIPT indication				
Routine screening/patient request	174 (57.4)	5 (20.8)	169 (60.6)	< 0.001
Advanced maternal age	58 (19.1)	12 (50.0)	46 (16.5)	
Abnormal first-trimester screening	32 (10.6)	3 (12.5)	29 (10.4)	
Ultrasound soft markers	28 (9.2)	3 (12.5)	25 (9.0)	
Previous affected pregnancy	7 (2.3)	1 (4.2)	6 (2.2)	
Other	4 (1.3)	0 (0)	4 (1.4)	
CapitalBio/Boao Proton	133 (43.9)	11 (45.8)	122 (43.7)	0.926
	103 (34.0)	8 (33.3)	95 (34.1)	
BGI MGISEQ-200	67 (22.1)	5 (20.8)	62 (22.2)	

Values are mean ± SD or n (%). BMI, body mass index; cffDNA, cell-free fetal DNA; DNA, deoxyribonucleic acid; GA, gestational age; NIPT, non-invasive prenatal testing.

### 3.2 Chromosomal abnormalities

Twenty-four pregnancies (7.9%; 95% CI: 5.4–11.5%) harbored confirmed chromosomal abnormalities (**Table 2**). Trisomy 21 was the most prevalent (n = 16, 66.7%), followed by trisomy 18 (n = 5, 20.8%), trisomy 13 (n = 2, 8.3%), and Turner syndrome (n = 1, 4.2%). The mean maternal age was significantly higher in the aneuploid group (37.4 ± 3.8 years vs. 30.4 ± 3.8 years; p < 0.001). The 7.9% prevalence exceeds that of unselected populations and reflects the enriched tertiary referral composition. The single case of Turner syndrome was included in the overall analyses; however, the study was not powered to evaluate sex chromosome aneuploidy detection independently.

**Table 2:** Distribution of confirmed chromosomal abnormalities (N = 24).

Diagnosis	n (%)	Mean maternal age (years)	Mean GA at diagnosis (weeks)
Trisomy 21	16 (66.7)	37.8 ± 3.9	20.6 ± 1.4
Trisomy 18	5 (20.8)	36.4 ± 4.2	20.2 ± 0.9
Trisomy 13	2 (8.3)	38.5 ± 2.1	21.0 ± 0.5
Turner syndrome (45,X)	1 (4.2)	34.0	20.5
Total	24 (100)	37.4 ± 3.8	20.6 ± 1.3

cffDNA, cell-free fetal DNA; DNA, deoxyribonucleic acid; GA, gestational age.

### 3.3 Diagnostic performance of NIPT alone

Of the 303 pregnancies, 43 (14.2%) had a positive NIPT result and 260 (85.8%) had a negative result (**Table 3**). All 24 chromosomal abnormalities were identified, yielding a sensitivity of 100% (95% CI: 86.2–100%) and an NPV of 100% (95% CI: 98.6–100%). As noted in Section 2.7, these estimates constitute upper bounds given reliance on neonatal phenotypic assessment for the NIPT-negative group. Nineteen false-positive results produced a specificity of 93.2% (260/279; 95% CI: 89.7–95.6%) and a PPV of 55.8% (24/43; 95% CI: 40.8–69.9%). The chromosome-specific PPV was 64.0% (16/25) for trisomy 21, 50.0% (5/10) for trisomy 18, and 40.0% (2/5) for trisomy 13. Individual-level data for all 43 NIPT-positive pregnancies, including NIPT platform, Z-score, chromosome flagged, ultrasound soft marker profile (type and count), karyotype result, and pregnancy outcome, are presented in **Supplementary Table S1**.

**Table 3:** NIPT performance and characteristics of true-positive versus false-positive results.

Parameter	True positive (n = 24)	False positive (n = 19)	p-value
NIPT Performance (N = 303)			
Sensitivity	100% (24/24; 95% CI: 86.2–100%)*		
Specificity	93.2% (260/279; 95% CI: 89.7–95.6%)		
PPV	55.8% (24/43; 95% CI: 40.8–69.9%)		
NPV	100% (260/260; 95% CI: 98.6–100%)*		
Characteristics			
Maternal age, years	37.4 ± 3.8	35.8 ± 4.6	0.213
BMI, kg/m²	25.2 ± 3.8	27.4 ± 4.2	0.081
Mean Z-score	28.6 ± 12.4	4.7 ± 1.1	< 0.001
Z-score range	8.2–56.3	3.2–6.5	—
Chromosome affected			
Chromosome 21	16 (66.7%)	9 (47.4%)	
Chromosome 18	5 (20.8%)	5 (26.3%)	
Chromosome 13	2 (8.3%)	3 (15.8%)	
Sex chromosomes	1 (4.2%)	2 (10.5%)	
Ultrasound soft markers			< 0.001
No markers	6 (25.0%)	12 (63.2%)	
1 marker	7 (29.2%)	7 (36.8%)	
≥ 2 markers	11 (45.8%)	0 (0%)	
Pregnancy outcome: live birth	5 (20.8%)	19 (100%)	< 0.001

*Upper-bound estimates given differential verification bias (see Methods Section 2.7). The PPV reported here represents the overall PPV across all chromosomal abnormalities; the chromosome-specific PPV for TS21 was 64.0% (16/25). CI, confidence interval; cffDNA, cell-free fetal DNA; DNA, deoxyribonucleic acid; NPV, negative predictive value; PPV, positive predictive value.

### 3.4 Characteristics of false-positive NIPT cases

The 19 false-positive cases demonstrated distinctive features compared to the 24 true positives (**Table 3**). The mean Z-scores were 4.7 ± 1.1 (range: 3.2–6.5), significantly lower than the 28.6 ± 12.4 (range: 8.2–56.3) observed among true positives (p < 0.001), indicating clustering within a borderline range. False-positive cases demonstrated a higher BMI (27.4 ± 4.2 kg/m²) compared to NIPT-negative euploid pregnancies (23.1 ± 2.8 kg/m²; p < 0.001). The affected chromosomes were chromosomes 21 (n = 9), 18 (n = 5), 13 (n = 3), and sex chromosomes (n = 2). Most pertinently, 12 of the 19 false-positive cases (63.2%) had no soft markers, 7 (36.8%) had a single marker, and none had ≥ 2 markers. All 19 were confirmed euploid by amniocentesis and resulted in phenotypically normal live births. While these findings are consistent with confined placental mosaicism (CPM) as the predominant underlying mechanism (29, 44), it should be acknowledged that CPM was inferred rather than directly confirmed in our cohort, as placental tissue was not systematically sampled or genotyped. As demonstrated by Li and Lai (45) in a detailed case involving a rare CPM, cffDNA in maternal blood originates primarily from apoptotic placental trophoblast cells and may not represent the actual fetal karyotype. Alternative explanations for NIPT false positives, including maternal copy number variants, low-level maternal mosaicism, co-twin demise in unrecognized vanishing twins, and technical artifacts, cannot be excluded. CPM rates vary by chromosome, with a higher prevalence for trisomies 13 and 18 than for trisomy 21, concordant with the proportionally elevated false-positive rates for these chromosomes observed in our cohort.

### 3.5 Prevalence and distribution of ultrasound soft markers

At least one soft marker was identified in 95 pregnancies (31.4%). Soft markers were significantly more prevalent among pregnancies that were aneuploid (18/24, 75.0%) than among euploid pregnancies (77/279, 27.6%; p < 0.001; OR = 7.87, 95% CI: 3.08–20.11) (**Table 4**). The mean marker count was 2.5 ± 2.5 in aneuploid versus 0.4 ± 0.7 in euploid pregnancies (p < 0.001).

**Table 4:** Prevalence of individual ultrasound soft markers and association with chromosomal abnormality.

Soft marker	All (N = 303)	Aneuploid (n = 24)	Euploid (n = 279)	p-value	OR (95% CI)
Any marker	95 (31.4)	18 (75.0)	77 (27.6)	< 0.001	7.87 (3.08–20.11)
High-risk markers					
Increased nuchal fold ≥ 6 mm	13 (4.3)	7 (29.2)	6 (2.2)	< 0.001†	18.89 (5.50–64.91)
Short femur	11 (3.6)	5 (20.8)	6 (2.2)	< 0.001†	11.86 (3.23–43.56)
Echogenic bowel	6 (2.0)	3 (12.5)	3 (1.1)	0.002†	13.00 (2.40–70.46)
Hypoplastic nasal bone	4 (1.3)	2 (8.3)	2 (0.7)	0.009	12.50 (1.65–94.69)
Short humerus	3 (1.0)	2 (8.3)	1 (0.4)	0.006	24.67 (2.12–286.6)
Low-risk markers					
EIF	54 (17.8)	7 (29.2)	47 (16.8)	0.134	2.03 (0.79–5.21)
CPC	34 (11.2)	7 (29.2)	27 (9.7)	0.005	3.84 (1.45–10.17)
Pyelectasis	27 (8.9)	5 (20.8)	22 (7.9)	0.036	3.08 (1.05–9.05)
By marker count				< 0.001	
0 markers	208 (68.6)	6 (25.0)	202 (72.4)		Reference
1 marker	69 (22.8)	7 (29.2)	62 (22.2)		3.80 (1.25–11.55)
2 markers	16 (5.3)	4 (16.7)	12 (4.3)		11.22 (2.90–43.38)
≥ 3 markers	10 (3.3)	7 (29.2)	3 (1.1)		78.50 (16.17–381.2)
Mean marker count	0.5 ± 0.8	2.5 ± 2.5	0.4 ± 0.7	< 0.001	—

Values are n (%) unless indicated. †Significant after Bonferroni correction (threshold p < 0.00625). cffDNA, cell-free fetal DNA; CI, confidence interval; CPC, choroid plexus cyst; DNA, deoxyribonucleic acid; EIF, echogenic intracardiac focus; OR, odds ratio.

Among the individual markers, high-risk markers exhibited the strongest discriminative capacity: increased nuchal fold ≥ 6 mm (OR = 18.89; 95% CI: 5.50–64.91), short humerus (OR = 24.67; 95% CI: 2.12–286.6), echogenic bowel (OR = 13.00; 95% CI: 2.40–70.46), hypoplastic nasal bone (OR = 12.50; 95% CI: 1.65–94.69), and short femur (OR = 11.86; 95% CI: 3.23–43.56). Low-risk markers exhibited comparatively modest associations (EIF: OR = 2.03; CPC: OR = 3.84; pyelectasis: OR = 3.08). After Bonferroni correction (adjusted threshold p < 0.00625), increased nuchal fold, short femur, and echogenic bowel retained statistical significance, whereas hypoplastic nasal bone and short humerus did not reach the corrected threshold owing to the rarity of these markers.

A dose-response relationship was observed: one marker (OR = 3.80; p = 0.019), two markers (OR = 11.22; p < 0.001), and ≥ 3 markers (OR = 78.50; p < 0.001).

### 3.6 Soft marker patterns by aneuploidy type

**Figure 2:**
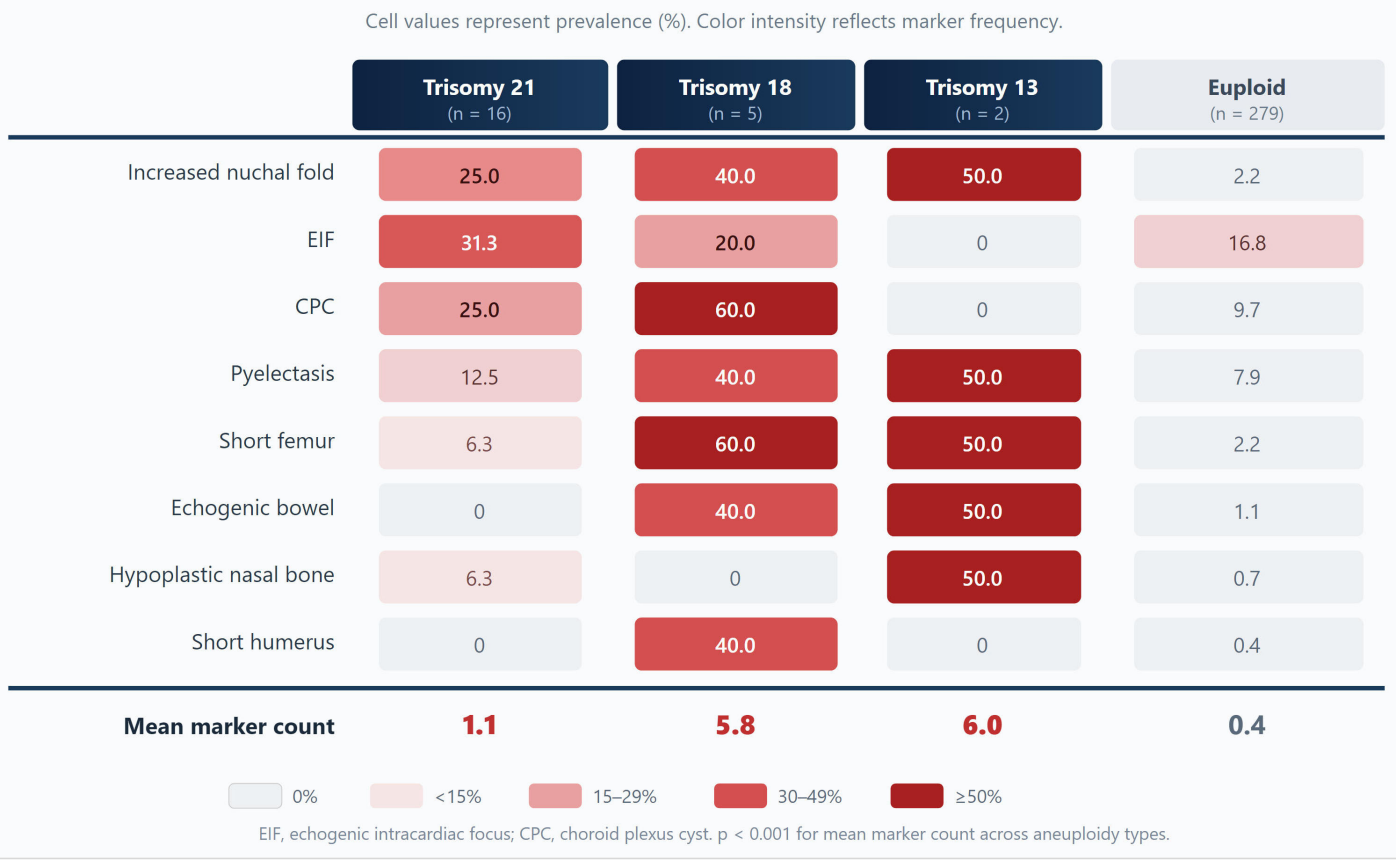
Heatmap of soft marker prevalence (%) across aneuploidy types and euploid pregnancies. Color intensity reflects marker frequency. Trisomy 18 and 13 demonstrate uniformly severe marker burdens, whereas trisomy 21 exhibits a characteristically subtle profile.

Phenotypic expression differed markedly across aneuploidy types (**Table 5**; **Figure 2**). Trisomy 21 (n = 16) exhibited a subtle profile (mean 1.1 ± 1.0 markers): 6/16 (37.5%) had no markers, and EIF (5/16, 31.3%), increased nuchal fold (4/16, 25.0%), and CPC (4/16, 25.0%) were most frequent; only 1/16 (6.3%) had ≥ 3 markers. Trisomy 18 (n = 5) demonstrated the most severe burden (mean 5.8 ± 1.3 markers), with all five cases exhibiting ≥ 3 markers. Short femur (3/5), CPC (3/5), increased nuchal fold (2/5), echogenic bowel (2/5), and short humerus (2/5) constituted the characteristic profile. Trisomy 13 (n = 2) showed similarly extensive involvement (mean 6.0 markers). The single Turner syndrome case had 2 markers. The difference in mean marker count across types was statistically significant (p < 0.001). Given the small subgroup sizes for trisomy 18, trisomy 13, and Turner syndrome, all percentages should be interpreted with appropriate caution.

**Table 5:** Soft marker profiles by chromosomal abnormality type.

Parameter	T21 (n = 16)	T18 (n = 5)	T13 (n = 2)	Turner (n = 1)	p-value
Mean markers (± SD)	1.1 ± 1.0	5.8 ± 1.3	6.0 ± 0.0	2.0	< 0.001
No markers, n (fraction)	6 (6/16)	0 (0/5)	0 (0/2)	0 (0/1)	0.079
≥ 3 markers, n (fraction)	1 (1/16)	5 (5/5)	2 (2/2)	0 (0/1)	< 0.001
Nuchal fold ≥ 6 mm	4 (4/16)	2 (2/5)	1 (1/2)	0	0.571
EIF	5 (5/16)	1 (1/5)	0 (0/2)	1 (1/1)	0.342
CPC	4 (4/16)	3 (3/5)	0 (0/2)	0	0.106
Short femur	1 (1/16)	3 (3/5)	1 (1/2)	0	0.006
Echogenic bowel	0 (0/16)	2 (2/5)	1 (1/2)	0	0.002
Short humerus	0 (0/16)	2 (2/5)	0 (0/2)	0	0.005

Given the small subgroup sizes for T18 (n = 5), T13 (n = 2), and Turner syndrome (n = 1), fractions are reported alongside percentages, and all values should be interpreted with caution. cffDNA, cell-free fetal DNA; CPC, choroid plexus cyst; DNA, deoxyribonucleic acid; EIF, echogenic intracardiac focus; T21, trisomy 21; T18, trisomy 18; T13, trisomy 13.

### 3.7 Diagnostic performance of combined NIPT–Ultrasound Strategies

**Figure 3:**
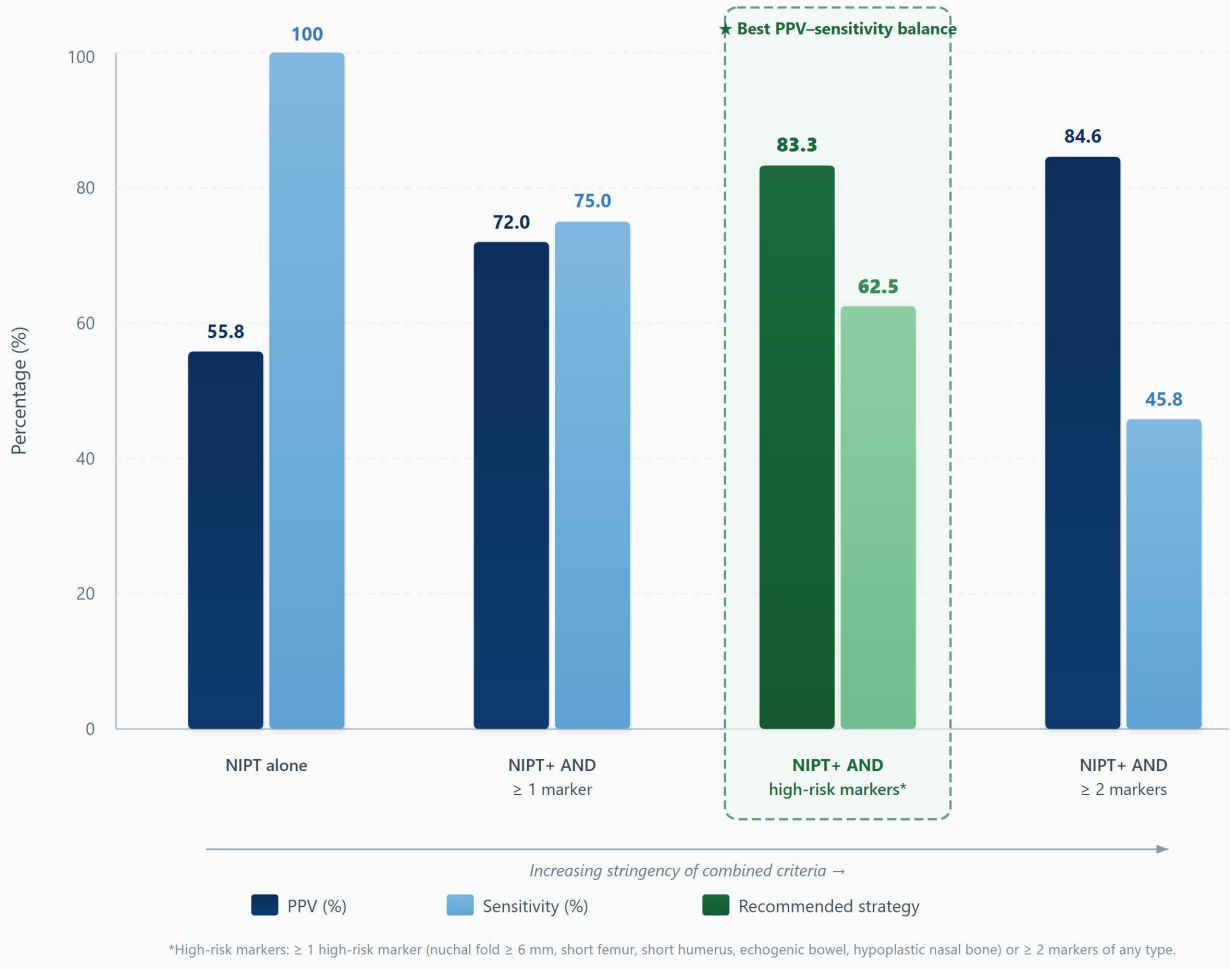
Trade-off between positive predictive value and sensitivity across combined NIPT–ultrasound screening strategies. The high-risk combined strategy achieved 83.3% PPV with 62.5% sensitivity, representing the most balanced approach for clinical implementation.

The four predefined combined strategies are compared with NIPT alone in **Table 6** and **Figure 3**. Strategy (a) (NIPT+ ≥ 1 marker) achieved a PPV of 72.0% (18/25; +16.2 pp versus NIPT alone) and specificity of 97.5%, but sensitivity declined to 75.0% as 6 marker-negative cases of trisomy 21 were missed. Strategy (b) (NIPT+ ≥ 2 markers) achieved the highest PPV (84.6%, 11/13) at a substantial sensitivity cost (45.8%). Strategy (c) (high-risk combined) provided the most balanced profile: sensitivity, 62.5%; specificity, 98.9%; PPV, 83.3% (15/18); and NPV, 96.8%. Strategy (d) (NIPT+ OR ≥ 2 markers), designed as a theoretical sensitivity benchmark rather than a practical clinical recommendation (see Section 2.5), preserved 100% sensitivity but eroded PPV to 22.9%, substantially increasing the number of recommended invasive procedures (NNT = 4.4). Among the 7 NIPT-positive cases without any markers, 6/7 (85.7%) were true positives, all with trisomy 21, demonstrating that NIPT positivity alone remains a potent predictor, even in the absence of sonographic abnormalities.

**Table 6:** Diagnostic performance of NIPT alone versus combined NIPT–ultrasound strategies.

Strategy	Sensitivity (%)	Specificity (%)	PPV (%)	NPV (%)	NNT†
NIPT alone	100 (24/24)	93.2 (260/279)	55.8 (24/43)	100 (260/260)	1.8
(a) NIPT+ AND ≥ 1 marker	75.0 (18/24)	97.5 (272/279)	72.0 (18/25)	97.8 (272/278)	1.4
(b) NIPT+ AND ≥ 2 markers	45.8 (11/24)	99.3 (277/279)	84.6 (11/13)	95.5 (277/290)	1.2
(c) NIPT+ AND high-risk markers*	62.5 (15/24)	98.9 (276/279)	83.3 (15/18)	96.8 (276/285)	1.2
(d) NIPT+ OR ≥ 2 markers	100 (24/24)	71.0 (198/279)	22.9 (24/105)	100 (198/198)	4.4
Ultrasound alone (≥ 1 marker)	75.0 (18/24)	72.4 (202/279)	18.9 (18/95)	97.1 (202/208)	5.3

*High-risk markers: ≥ 1 high-risk marker (nuchal fold ≥ 6 mm, short femur, short humerus, echogenic bowel, hypoplastic nasal bone) or ≥ 2 markers of any type. †cffDNA, cell-free fetal DNA; DNA, deoxyribonucleic acid; NNT, number of invasive tests per one confirmed abnormality; NPV, negative predictive value; PPV, positive predictive value.

### 3.8 Aneuploidy-type-specific performance of combined strategies

The performance of the combined strategies differed substantially by aneuploidy type (**Table 7**). For trisomy 18 and 13 combined (n = 7), Strategy (a) achieved 100% sensitivity (7/7) and 100% PPV, as all cases harbored multiple markers. For trisomy 21 (n = 16), Strategy (a) achieved 62.5% sensitivity (10/16) because 6 cases lacked any markers. Strategy (c) similarly achieved 100% sensitivity for trisomies 18 and 13 but only 50.0% for trisomy 21 (8/16). These data indicate that the combined approach functions as a near-confirmatory tool for trisomies 18 and 13 and as a risk-stratification tool for trisomy 21, where phenotypic heterogeneity necessitates continued reliance on NIPT positivity as the primary risk indicator. Accordingly, the overall PPV improvement reported for the combined strategies should be interpreted with the caveat that the benefit is not uniform across aneuploidy types and is substantially less impactful for trisomy 21, the most clinically prevalent scenario.

**Table 7:** Aneuploidy-type-specific performance of combined strategies.

Aneuploidy type	n	Cases with ≥ 1 marker	Strategy (a) sensitivity	Strategy (c) sensitivity	Notes
Trisomy 21	16	10/16	62.5% (10/16)	50.0% (8/16)	6 cases marker-negative
Trisomy 18	5	5/5	100% (5/5)	100% (5/5)	All had ≥ 3 markers
Trisomy 13	2	2/2	100% (2/2)	100% (2/2)	All had ≥ 3 markers
Turner syndrome	1	1/1	100% (1/1)	100% (1/1)	2 markers present
T18 + T13 combined	7	7/7	100% (7/7)	100% (7/7)	100% concordance
Total	24	18/24	75.0% (18/24)	62.5% (15/24)	All missed: T21

Strategy (a), NIPT+ AND ≥ 1 marker. Strategy (c), NIPT+ AND high-risk markers (≥ 1 high-risk marker or ≥ 2 any markers). Combined strategies achieve near-confirmatory accuracy for trisomies 18 and 13 but have reduced sensitivity for trisomy 21 owing to phenotypic heterogeneity. cffDNA, cell-free fetal DNA; DNA, deoxyribonucleic acid.

### 3.9 Impact of maternal age on combined screening performance

Age-stratified analysis revealed differential benefits (**Table 8**). In women aged < 35 years (n = 239), NIPT PPV was 33.3%, and strategy (a) improved PPV to 61.5% (+28.2 pp). In women aged ≥ 35 years (n = 64), NIPT PPV was already 84.2%, and strategy (a) yielded 91.7% (+7.5 pp). Strategy (a) was selected for the age-stratified analysis as the simplest binary criterion permitting stable subgroup estimation. These results should be interpreted as exploratory, given the limited events per subgroup (8 and 16 aneuploid cases, respectively).

**Table 8:** NIPT and combined screening performance stratified by maternal age.

Age group	N	Aneuploid, n (%)	NIPT PPV (%)	Combined PPV* (%)	Absolute gain (pp)
< 35 years	239	8 (3.3)	33.3 (8/24)	61.5 (8/13)	+28.2
≥ 35 years	64	16 (25.0)	84.2 (16/19)	91.7 (11/12)	+7.5
p-value	—	< 0.001	< 0.001	0.048	—

*Combined = Strategy (a), NIPT-positive and ≥ 1 marker. Strategy (a) was selected as the simplest binary criterion for stable age-stratified estimation. Results are exploratory. cffDNA, cell-free fetal DNA; DNA, deoxyribonucleic acid; pp, percentage points; PPV, positive predictive value.

### 3.10 Impact on invasive testing utilization

If Strategy (c) is adopted as a criterion for prioritizing the strength of invasive testing recommendations within a shared decision-making framework, the number of procedures receiving strong recommendations would be reduced from 43 to 18 (58% reduction), detecting 15/24 abnormalities with three false positives and a number needed to test (NNT) of 1.2. The remaining 9 abnormalities (all trisomy 21 without high-risk markers) would still be identified through NIPT positivity and offered genetic counseling with the option of invasive testing. The combined strategy thus functions not as a gatekeeper restricting diagnostic access; it should be emphasized that all patients with positive NIPT should continue to be offered invasive diagnostic testing in accordance with current clinical guidelines, including ACOG Practice Bulletin No. 226 (13) and SMFM Consult #57 (15), but as a risk-stratification instrument enabling differentiated counseling: pregnancies with concordant molecular and sonographic findings would proceed directly to invasive testing with a strong recommendation, while discordant findings (NIPT-positive without markers) would receive counseling regarding the elevated false-positive probability and contingent options, including repeat NIPT, expanded panel testing, or serial ultrasound surveillance.

### 3.11 Logistic regression analysis

**Figure 4:**
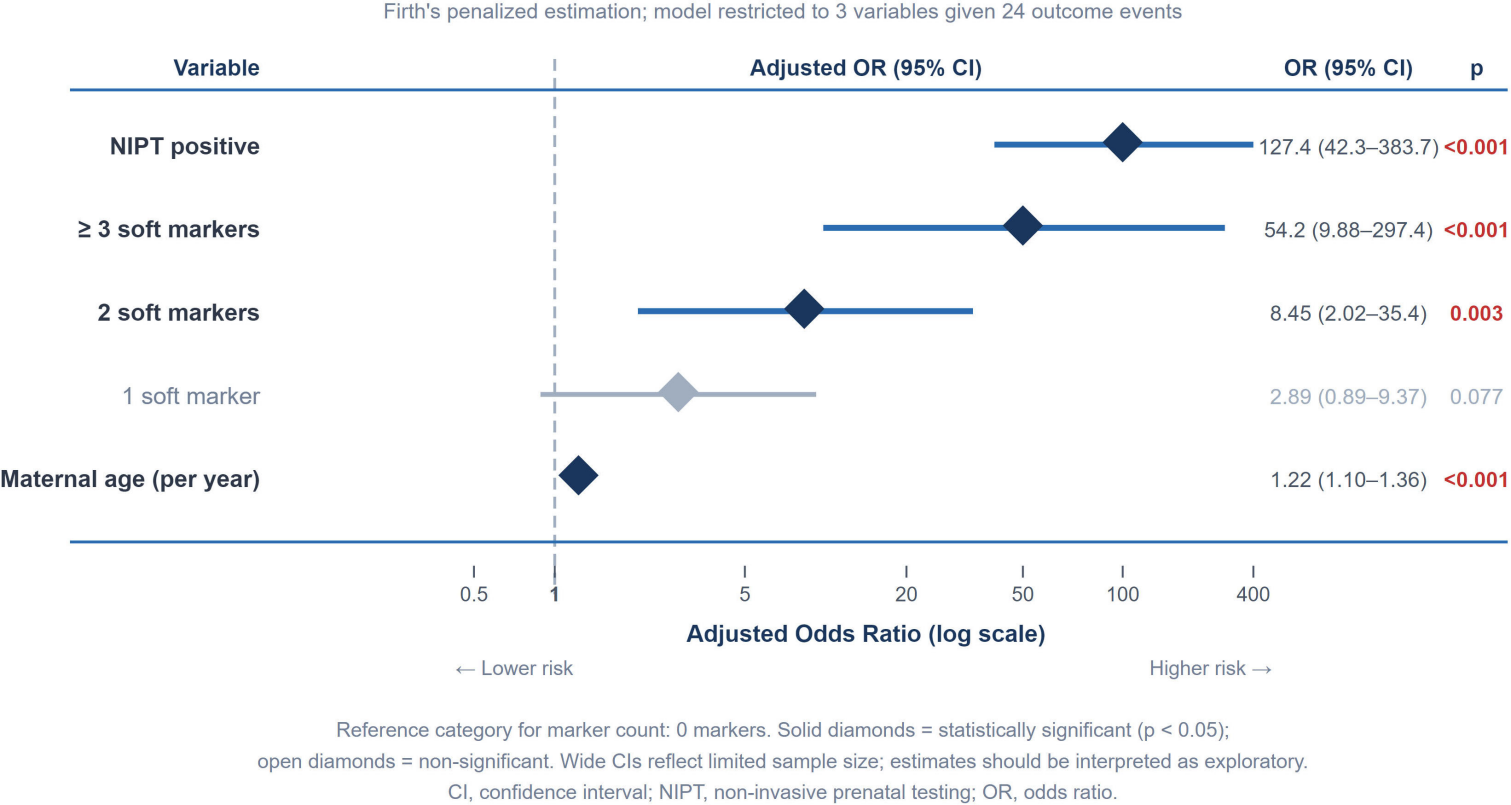
Forest plot of adjusted odds ratios with 95% confidence intervals from multivariable logistic regression (Firth’s penalized estimation). The model was restricted to three variables, given 24 outcome events. Wide confidence intervals reflect the limited sample size.

Multivariable logistic regression using Firth’s penalized estimation identified independent predictors of chromosomal abnormalities (**Table 9**; **Figure 4**). NIPT positivity was the strongest predictor (adjusted OR = 127.4; 95% CI: 42.3–383.7; p < 0.001); this estimate is influenced by quasi-complete separation and indicates an extremely strong association rather than a precise effect magnitude. The number of soft markers demonstrated an independent dose-dependent association: 2 markers (adjusted OR = 8.45; 95% CI: 2.02–35.37; p = 0.003) and ≥ 3 markers (adjusted OR = 54.22; 95% CI: 9.88–297.4; p < 0.001). Increasing maternal age contributed incrementally (adjusted OR = 1.22 per year; 95% CI: 1.10–1.36; p < 0.001). Wide confidence intervals reflect the limited sample size; therefore, these estimates should be interpreted as exploratory.

**Table 9:** Multivariable logistic regression for independent predictors of chromosomal abnormality (firth’s penalized estimation).

Variable	Adjusted OR (95% CI)	p-value
NIPT positive	127.4 (42.3–383.7)*	< 0.001
Number of soft markers (vs. 0)		
1 marker	2.89 (0.89–9.37)	0.077
2 markers	8.45 (2.02–35.37)	0.003
≥ 3 markers	54.22 (9.88–297.4)	< 0.001
Maternal age (per year)	1.22 (1.10–1.36)	< 0.001

*Boundary estimate influenced by quasi-complete separation; indicates direction and strength of association rather than precise effect magnitude. The model was restricted to three variables, given 24 outcome events (~8 events per variable). Model AUC: 0.987 (95% CI: 0.974–1.000). Wide confidence intervals reflect a limited sample size; therefore, estimates should be interpreted as exploratory. cffDNA, cell-free fetal DNA; CI, confidence interval; DNA, deoxyribonucleic acid; OR, odds ratio.

### 3.12 ROC curve analysis

**Figure 5:**
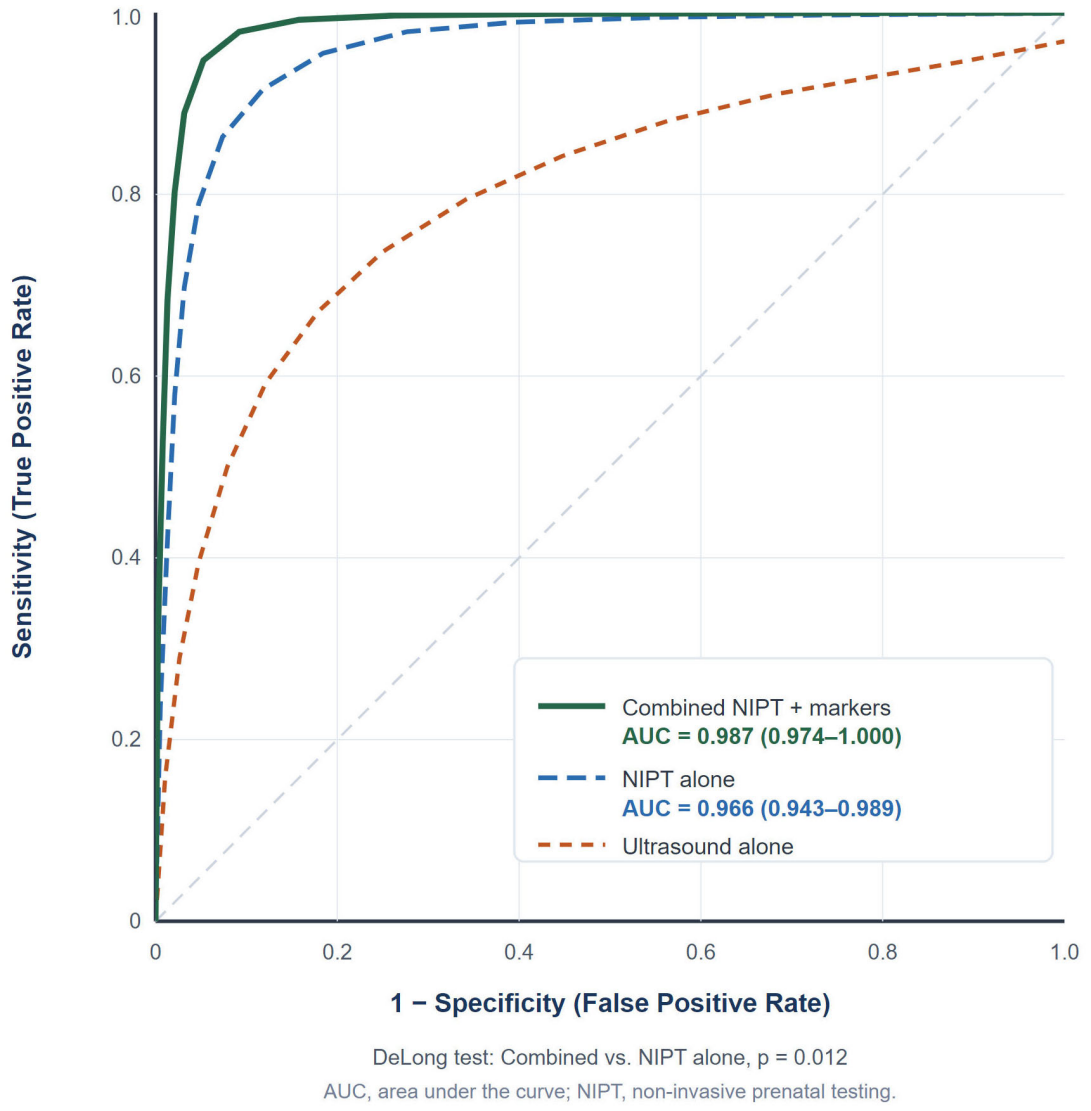
Receiver operating characteristic curves comparing NIPT alone (AUC = 0.966), ultrasound markers alone (AUC = 0.877), and the combined NIPT plus marker count model (AUC = 0.987). The combined model was significantly superior to NIPT alone (p = 0.012, DeLong test).

The combined NIPT plus marker count model achieved the highest AUC (0.987; 95% CI: 0.974–1.000), significantly exceeding that of NIPT alone (AUC = 0.966; p = 0.012 by DeLong test; **Figure 5**). Ultrasound markers alone yielded an AUC of 0.877. The combined NIPT plus high-risk marker model achieved an AUC of 0.982 (p = 0.024 versus NIPT alone).

### 3.13 Pregnancy outcomes

Of the 24 aneuploid pregnancies, 18 (75.0%) underwent elective termination at a mean of 21.4 ± 1.8 weeks, 5 (20.8%) resulted in live birth (3 with Trisomy 21, 1 with Turner syndrome, and 1 with late-diagnosed Trisomy 21), and 1 case of Trisomy 18 (4.2%) resulted in stillbirth at 28 weeks. All 19 false-positive and 260 true-negative pregnancies resulted in phenotypically normal live births.

## 4 Discussion

This three-year retrospective cohort study provides preliminary evidence suggesting that integrating second-trimester ultrasound soft markers with NIPT may improve the positive predictive value for fetal aneuploidy detection in an enriched tertiary referral cohort. Although NIPT alone identified all 24 confirmed chromosomal abnormalities, its PPV of 55.8% signified that nearly half of all positive results were false positives, a proportion with tangible consequences in terms of parental anxiety and unnecessary invasive procedures. The high-risk combined strategy raised the PPV to 83.3% with 98.9% specificity and only 3 false positives among 18 recommended procedures, while reducing invasive testing by 58%. These findings, if confirmed in larger prospective studies with populations of varying aneuploidy prevalence, support a risk-stratified clinical paradigm in which ultrasound findings refine post-test probability following a positive NIPT result rather than supplanting molecular screening itself. Importantly, the observed PPV improvement was predominantly driven by trisomies 18 and 13, which uniformly presented with multiple ultrasound markers, whereas the combined approach demonstrated more limited utility for trisomy 21, where 37.5% of cases were marker-negative. In a large-scale Chinese study of 59,800 pregnancies, Wang et al. (46) demonstrated that pregnancies with ultrasound soft-marker abnormalities had significantly higher PPVs than other clinical subgroups (p = 0.01). The very high-performance metrics in our study (e.g., AUC = 0.987) should be interpreted with caution, as they may reflect optimism bias; external validation in independent cohorts is necessary to confirm these discriminative properties. Importantly, this study addresses a clinical scenario employing ultrasound to refine risk after positive NIPT that is distinct from and complementary to the framework established by SMFM Consult #57 (15), which addresses management of soft markers identified after reassuring screening.

The observed NIPT performance 100% sensitivity (acknowledging the upper-bound nature of this estimate given differential verification bias) and 93.2% specificity aligns with international (9–11) and Chinese validation studies (18, 31). The PPV of 55.8%, falling at the lower end of published ranges, is attributable to the demographic composition of our cohort, in which 78.9% of women were younger than 35 years with correspondingly lower baseline aneuploidy prevalence. This finding exemplifies a principle frequently underappreciated in clinical practice: PPV is critically dependent on disease prevalence, such that even a highly specific test generates a disproportionate number of false positives when applied to low-prevalence populations (16, 17). Zhang et al. (18) similarly reported that NIPT PPV for trisomy 21 was substantially higher among high-risk subgroups than among voluntary screening participants (56.73% versus 32.81%) in a Beijing cohort of 68,763 pregnancies, and Kagan et al. (47) recently demonstrated that age-stratified combined PPV ranges from 48.7% to 98.8% across the maternal age spectrum. By Bayesian extrapolation, the incremental benefit of our combined approach is anticipated to be even greater in unselected populations with lower baseline prevalence, although this projection assumes stable specificity across prevalence settings and warrants prospective validation. Notably, the false-positive cases demonstrated Z-scores clustering within the 3.0–7.0 range (mean 4.7 ± 1.1), whereas true positives exhibited substantially higher values (mean 28.6 ± 12.4). This quantitative distinction, in conjunction with ultrasound marker status, represents a hypothesis-generating observation that warrants prospective validation; given the retrospective design, limited sample size, platform heterogeneity, and incomplete fetal fraction data, these findings should not be interpreted as a basis for clinical decision-making at this stage. Spingler et al. (26) similarly identified fetal fraction as a significant contributor to true versus false positive cfDNA results. Individual-level fetal fraction data were not uniformly available across the two NIPT platforms; this represents a priority for future investigation.

The false-positive cases in our cohort exhibited a constellation of features with direct clinical implications: borderline Z-scores (mean 4.7 versus 28.6 in true positives; p < 0.001), elevated BMI, and most pertinently, phenotypic discordance with molecular findings. The observation that 63.2% of false positives had no soft markers and none had ≥ 2 markers provides a mechanistic rationale for the combined approach: CPM, wherein chromosomal aberrations exist in trophoblastic tissue (the cffDNA source) but not in the fetus (29), produces molecular positivity without corresponding sonographic abnormalities. CPM rates are known to vary by chromosome, with a higher prevalence for trisomies 13 and 18 (29), consistent with the proportionally elevated false-positive rates for these chromosomes in our data. Recent studies provide instructive contrasts. Gug et al. (48) reported 100% confirmation of NIPT-detected trisomy 21 risk in a Romanian cohort of 380 pregnancies, and Gug et al. (49) similarly achieved 100% confirmation in 15 trisomy 21 cases among 1,400 pregnancies. These perfect confirmation rates contrast with our chromosome 21-specific PPV of 64.0%, a discrepancy attributable to differences in cohort composition and baseline prevalence, illustrating the Bayesian dependence of PPV on pretest probability. Our findings also corroborate those of Spingler et al. (26), who reported that the PPV for trisomy 18/13 declined to 9.5% when ultrasound was normal but reached 100% when abnormalities were present. The substantially higher pre-ultrasound PPV for trisomy 21 in that study (98.3% versus our 55.8%) reflects their older median maternal age (37.7 years) and tertiary referral composition, underscoring that the absolute magnitude of PPV improvement from ultrasound integration depends on the baseline PPV determined by population characteristics.

The diagnostic utility of soft markers exhibited a clear dose-response relationship, escalating from an OR of 3.80 for one marker to an OR of 78.50 for three or more markers. High-risk markers, particularly the increased nuchal fold, shortened long bones, and echogenic bowel, demonstrated the strongest associations, consistent with the likelihood ratio hierarchy described by the SMFM (15) and classical literature (22–24, 38, 50). The “genetic sonogram” concept, which synthesizes multiple marker assessments to modify the *a priori* risk of trisomy 21, provided the conceptual foundation for integrating marker profiles into aneuploidy risk estimation (50). Conversely, isolated EIF (51), CPC, and pyelectasis exhibited modest discriminative value (ORs 2.03–3.84), corroborating the consensus that these isolated findings warrant no further invasive evaluation after reassuring NIPT (52). Our data extend this principle to the post-positive NIPT scenario: all 7 false-positive cases with a single marker harbored exclusively low-risk markers, reinforcing their limited discriminative capacity, even when NIPT is positive. A consideration of particular relevance to our Chinese cohort concerns the population-specific applicability of hypoplastic nasal bone as a soft marker. Nasal bone hypoplasia is more prevalent as a normal anatomical variant among individuals of East Asian descent, with the rate of absent nasal bone in euploid fetuses approximately threefold higher in East Asians than in European populations (53, 54). In our cohort, hypoplastic nasal bone was observed in only 1 of 16 (6.3%) trisomy 21 cases and 2 of 279 (0.7%) euploid pregnancies; the resulting odds ratio (12.50; 95% CI: 1.65–94.69) did not retain significance after Bonferroni correction. These data indicate that ethnic-specific normative data should be considered when interpreting nasal bone assessments in Chinese populations. This consideration does not diminish the overall utility of the combined screening approach, as four additional high-risk markers demonstrated robust discriminative capacity.

The pronounced phenotypic heterogeneity across aneuploid types constitutes a finding of considerable clinical salience. Trisomy 21 exhibited a characteristically subtle profile (mean 1.1 markers; 37.5% marker-negative), whereas trisomy 18 and 13 demonstrated uniformly severe burdens (mean 5.8 and 6.0 markers, respectively; 100% with ≥ 3 markers). This dichotomy translates directly into the differential performance of combined strategies: 100% sensitivity and PPV for trisomies 18 and 13 versus 50–62.5% sensitivity for trisomy 21. The clinical implication is immediate: a normal second trimester ultrasound following a positive NIPT for trisomy 18 or 13 substantially diminishes the post-test probability and may be meaningfully incorporated into shared decision-making regarding invasive procedures, whereas a normal ultrasound following a positive NIPT for trisomy 21 affords considerably less reassurance, and invasive testing should continue to be offered.

The age-stratified analysis revealed that younger women derived the greatest incremental benefit (+28.2 points [pp] in women < 35 years versus +7.5 pp in women ≥ 35 years), consistent with the Bayesian principle that adjunctive tests are most informative when the pretest probability is lowest [16]. This observation suggests that age-stratified implementation may be particularly warranted for younger NIPT-positive women, who could be counseled regarding the elevated false-positive probability and offered contingent options before proceeding to invasive testing. An important practical consideration pertains to the temporal sequence: NIPT positivity is typically available before 16 weeks, whereas a detailed ultrasound occurs at 19–23 weeks. The proposed approach therefore requires counseling NIPT-positive patients about the potential value of incorporating ultrasound findings into the risk assessment without restricting access to invasive procedures for those who prefer immediate diagnostic clarity. Ultrasound findings, when available, either reinforce the indication for invasive testing (concordant findings) or modulate the post-test probability (discordant findings), thereby supporting more informed decision-making rather than serving as a gatekeeper.

These findings complement and extend the existing guideline framework. SMFM Consult #57 (15) appropriately downgrades isolated soft markers after negative NIPT; the SOGC Guideline No. 456 (55) acknowledges the second-trimester ultrasound as a valuable adjunct in high-risk contexts; and the Kagan group (47) has recently affirmed that soft markers retain relevance for refining risk beyond common trisomy screening. Our study provides empirical data for the post-positive NIPT scenario that these guidelines do not directly address and suggests that the structured integration of ultrasound data into post-positive NIPT counseling warrants consideration in future guideline iterations. From an ethical standpoint, it should be emphasized that the combined approach is intended to enhance counseling rather than restrict diagnostic access; all patients with positive NIPT should continue to be offered invasive testing regardless of ultrasound findings.

The present study has several methodological strengths: inclusion of all consecutive eligible pregnancies over 36 months, complete outcome data, excellent inter-rater reliability (kappa = 0.94), real-world multi-platform NIPT data, predefined strategy classification, and aneuploidy-type-specific analysis. The universal provision of second-trimester ultrasound within Chinese prenatal care permits implementation without additional cost or procedural burden. However, important limitations warrant consideration. The retrospective single-center tertiary design limits generalizability to community settings, where operator experience may vary. The 7.9% aneuploidy prevalence reflects cohort enrichment; therefore, PPV estimates will differ in unselected populations. Verification bias from reliance on neonatal phenotypic assessment in NIPT-negative pregnancies may have inflated sensitivity and NPV, and the 100% values should be interpreted as upper bounds. Small subgroup sizes for trisomy 18 (n = 5), trisomy 13 (n = 2), and Turner syndrome (n = 1) limit the precision of type-specific analyses. Individual-level fetal fraction data were not uniformly available across platforms, precluding a formal comparative analysis between true-positive and false-positive cases, which would have strengthened the mechanistic interpretation. Inter-observer variability in soft marker assessment was not formally evaluated. The exclusion of single umbilical artery and mild ventriculomegaly may have underestimated the marker burden in certain cases. Logistic regression confidence intervals are wide and should be considered exploratory. The very high AUC value (0.987) may reflect optimism bias arising from the limited sample size and enriched cohort; external validation in independent datasets is essential. Variability in bioinformatic processing, fetal fraction handling, and result interpretation across the three NIPT platforms may influence false-positive rates in ways that our pooled analysis cannot fully capture; platform-stratified analysis should be prioritized in future multicenter studies. Finally, a formal cost effectiveness analysis was not performed.

Future investigations should prioritize prospective multicenter validation with predefined protocols and universal karyotyping to eliminate verification bias. A formal health economic evaluation, particularly regarding cost savings from reduced unnecessary amniocenteses, is needed. Integration of Z-score magnitude into risk algorithms may further refine discrimination, as our data suggest that borderline Z-scores (3.0–7.0) combined with the absence of markers may characterize a high-probability false-positive subgroup; however, this observation remains hypothesis-generating and requires validation in larger, prospectively designed cohorts before clinical application. Machine learning approaches incorporating quantitative NIPT parameters, ultrasound measurements, fetal fraction, and maternal demographics represent a promising avenue for individualized risk prediction.

## 5 Conclusions

This study provides preliminary evidence suggesting that integrating second-trimester ultrasound soft markers with NIPT may improve the positive predictive value for fetal aneuploidy detection in an enriched Chinese tertiary referral cohort. Although NIPT alone achieved complete detection within the constraints of the reference standard (noting that the reported 100% sensitivity and 100% NPV represent upper-bound estimates given differential verification bias), its PPV of 55.8% engenders a considerable false-positive burden. The combined approach, incorporating NIPT positivity with high-risk ultrasound markers, elevates PPV to 83.3%, while reducing invasive procedures by 58%. Distinct phenotypic profiles subtle in trisomy 21, severe in trisomies 18 and 13, provide clinically actionable information, with the combined approach achieving near-confirmatory accuracy for trisomies 18 and 13. Importantly, the observed PPV improvement is not uniform across aneuploidy types; the combined approach is substantially less impactful for trisomy 21, the most common clinical scenario, owing to its phenotypic heterogeneity. The greatest incremental benefit accrues to younger women in whom baseline NIPT PPV is suboptimal. Given the universal availability of detailed second-trimester ultrasonography within Chinese prenatal care, this integrated approach can be implemented without additional cost. These findings, while preliminary, complement existing guideline frameworks and support further investigation of incorporating second-trimester ultrasound data into post-positive NIPT counseling. Prospective multicenter validation in populations with varying aneuploidy prevalence is essential before consideration for implementation into clinical protocols.

## Data Availability

The raw data supporting the conclusions of this article will be made available by the authors, without undue reservation.
