# Supplementary material for: Improved positive predictive value of non-invasive prenatal testing through integration with second-trimester ultrasound soft markers for fetal chromosomal abnormalities: a retrospective cohort study
Source: Front Endocrinol (Lausanne). 2026 May 29;17:1831332. doi: 10.3389/fendo.2026.1831332 (PMC13259757; doi:10.3389/fendo.2026.1831332)
Supplement: Supplementary file 1 [file Table1.docx]

**Supplementary Table S1. Individual-Level Data for All NIPT-Positive Pregnancies (N = 43)**

| **Case** | **NIPT Platform** | **Chr** | **Z-score** | **NF** | **EIF** | **CPC** | **Pyel** | **SF** | **SH** | **EB** | **HNB** | **Count** | **Karyotype** | **Outcome** |
| --- | --- | --- | --- | --- | --- | --- | --- | --- | --- | --- | --- | --- | --- | --- |
| **True Positive Cases (n = 24)** | | | | | | | | | | | | | | |
| 1 | BGI-NIFTY | 21 | 42.1 |  |  |  |  |  |  |  |  | **0** | 47,XX,+21 | TOP |
| 2 | BGI-NIFTY | 21 | 38.5 |  |  |  |  |  |  |  |  | **0** | 47,XY,+21 | TOP |
| 3 | Berry NIPT-seq | 21 | 35.2 |  |  |  |  |  |  |  |  | **0** | 47,XX,+21 | TOP |
| 4 | Harmony | 21 | 29.8 |  |  |  |  |  |  |  |  | **0** | 47,XY,+21 | Live birth |
| 5 | BGI-NIFTY | 21 | 26.3 |  |  |  |  |  |  |  |  | **0** | 47,XX,+21 | Live birth |
| 6 | Berry NIPT-seq | 21 | 22.7 |  |  |  |  |  |  |  |  | **0** | 47,XY,+21 | Live birth |
| 7 | BGI-NIFTY | 21 | 34.6 | 1 |  |  |  |  |  |  |  | **1** | 47,XX,+21 | TOP |
| 8 | Berry NIPT-seq | 21 | 31.4 | 1 |  |  |  |  |  |  |  | **1** | 47,XY,+21 | TOP |
| 9 | BGI-NIFTY | 21 | 27.9 |  | 1 |  |  |  |  |  |  | **1** | 47,XX,+21 | TOP |
| 10 | Harmony | 21 | 20.1 |  | 1 |  |  |  |  |  |  | **1** | 47,XY,+21 | TOP |
| 11 | BGI-NIFTY | 21 | 18.4 |  | 1 |  |  |  |  |  |  | **1** | 47,XX,+21 | TOP |
| 12 | Berry NIPT-seq | 21 | 15.9 |  |  | 1 |  |  |  |  |  | **1** | 47,XY,+21 | TOP |
| 13 | BGI-NIFTY | 21 | 12.3 |  |  |  | 1 |  |  |  |  | **1** | 47,XX,+21 | Live birth |
| 14 | Berry NIPT-seq | 21 | 56.3 | 1 |  | 1 |  |  |  |  |  | **2** | 47,XY,+21 | TOP |
| 15 | Harmony | 21 | 8.2 |  | 1 | 1 |  |  |  |  |  | **2** | 47,XX,+21 | TOP |
| 16 | BGI-NIFTY | 21 | 24.8 | 1 | 1 | 1 | 1 | 1 |  |  | 1 | **6** | 47,XY,+21 | TOP |
| 17 | Berry NIPT-seq | 18 | 36.7 | 1 |  | 1 | 1 | 1 |  | 1 |  | **5** | 47,XY,+18 | TOP |
| 18 | BGI-NIFTY | 18 | 28.3 |  | 1 | 1 |  | 1 | 1 | 1 |  | **5** | 47,XX,+18 | TOP |
| 19 | Harmony | 18 | 41.2 | 1 |  | 1 | 1 | 1 | 1 | 1 |  | **6** | 47,XY,+18 | TOP |
| 20 | BGI-NIFTY | 18 | 33.5 | 1 | 1 |  | 1 | 1 | 1 | 1 |  | **6** | 47,XX,+18 | Stillbirth |
| 21 | Berry NIPT-seq | 18 | 25.9 | 1 |  | 1 | 1 | 1 | 1 | 1 | 1 | **7** | 47,XY,+18 | TOP |
| 22 | BGI-NIFTY | 13 | 19.4 | 1 |  |  | 1 | 1 | 1 | 1 | 1 | **6** | 47,XX,+13 | TOP |
| 23 | Berry NIPT-seq | 13 | 22.8 | 1 |  |  | 1 | 1 | 1 | 1 | 1 | **6** | 47,XY,+13 | TOP |
| 24 | Harmony | X | N/A |  | 1 |  | 1 |  |  |  |  | **2** | 45,X | Live birth |
| **False Positive Cases (n = 19)** | | | | | | | | | | | | | | |
| 25 | BGI-NIFTY | 21 | 4.2 |  |  |  |  |  |  |  |  | **0** | 46,XX | Live birth |
| 26 | Berry NIPT-seq | 21 | 5.1 |  |  |  |  |  |  |  |  | **0** | 46,XY | Live birth |
| 27 | BGI-NIFTY | 21 | 3.8 |  |  |  |  |  |  |  |  | **0** | 46,XX | Live birth |
| 28 | Harmony | 21 | 4.9 |  |  |  |  |  |  |  |  | **0** | 46,XY | Live birth |
| 29 | BGI-NIFTY | 21 | 3.5 |  |  |  |  |  |  |  |  | **0** | 46,XX | Live birth |
| 30 | Berry NIPT-seq | 21 | 5.8 |  | 1 |  |  |  |  |  |  | **1** | 46,XY | Live birth |
| 31 | BGI-NIFTY | 21 | 4.6 |  |  | 1 |  |  |  |  |  | **1** | 46,XX | Live birth |
| 32 | Berry NIPT-seq | 21 | 6.2 |  | 1 |  |  |  |  |  |  | **1** | 46,XY | Live birth |
| 33 | Harmony | 21 | 3.9 |  |  |  | 1 |  |  |  |  | **1** | 46,XX | Live birth |
| 34 | BGI-NIFTY | 18 | 4.3 |  |  |  |  |  |  |  |  | **0** | 46,XY | Live birth |
| 35 | Berry NIPT-seq | 18 | 5.4 |  |  |  |  |  |  |  |  | **0** | 46,XX | Live birth |
| 36 | BGI-NIFTY | 18 | 3.2 |  |  |  |  |  |  |  |  | **0** | 46,XY | Live birth |
| 37 | Harmony | 18 | 6.5 |  |  | 1 |  |  |  |  |  | **1** | 46,XX | Live birth |
| 38 | Berry NIPT-seq | 18 | 4.8 |  | 1 |  |  |  |  |  |  | **1** | 46,XY | Live birth |
| 39 | BGI-NIFTY | 13 | 3.7 |  |  |  |  |  |  |  |  | **0** | 46,XX | Live birth |
| 40 | Berry NIPT-seq | 13 | 5.3 |  |  |  |  |  |  |  |  | **0** | 46,XY | Live birth |
| 41 | Harmony | 13 | 4.1 |  |  |  | 1 |  |  |  |  | **1** | 46,XX | Live birth |
| 42 | BGI-NIFTY | X | N/A |  |  |  |  |  |  |  |  | **0** | 46,XX | Live birth |
| 43 | Berry NIPT-seq | X/Y | N/A |  |  |  |  |  |  |  |  | **0** | 46,XY | Live birth |

cffDNA, cell-free fetal DNA; Chr, chromosome flagged by NIPT; CPC, choroid plexus cyst; DNA, deoxyribonucleic acid; EB, echogenic bowel; EIF, echogenic intracardiac focus; HNB, hypoplastic nasal bone; NF, increased nuchal fold (≥ 6 mm); NIPT, non-invasive prenatal testing; Pyel, pyelectasis; SF, short femur; SH, short humerus; TOP, termination of pregnancy. Soft marker presence is indicated by 1; absence by blank cell. Count is the sum of all eight markers. Z-scores are reported where available from archived platform records; N/A indicates data not available for sex chromosome analysis.

Cases 1–24 represent true positive results (abnormal karyotype confirmed by amniocentesis). Cases 25–43 represent false positive results (normal karyotype confirmed by amniocentesis; all resulted in phenotypically normal live births). Grey shading indicates false positive cases.
